# Intramuscular Dissecting Baker’s Cysts: A Case Series Highlighting Ultrasound Diagnosis and Differential Considerations

**DOI:** 10.7759/cureus.53658

**Published:** 2024-02-05

**Authors:** Delange Augustin, Delange Hendrick Augustin, Almenord Pharol, Valerie Deverson, Clifford Georges Patrick Khawly

**Affiliations:** 1 Radiology, Hôpital de l'Université d'Etat d'Haïti, Port-au-Prince, HTI; 2 Orthopaedics and Traumatology, Hôpital Universitaire la Paix, Port-au-Prince, HTI; 3 Radiology, Hôpital de l’Université d’Etat d’Haiti, Port-au-Prince, HTI

**Keywords:** differential diagnosis, ultrasound diagnosis, gastrocnemius, intramuscular, baker's cyst

## Abstract

Baker’s cysts, commonly incidental findings, can occasionally present as intramuscular dissecting cysts within the medial gastrocnemius muscle. This case report highlights the ultrasound features and differential diagnoses of intramuscular dissecting Baker’s cysts through the examination of three distinct cases: a 64-year-old woman with severe osteoarthritis, an 80-year-old man with a palpable mass in the popliteal fossa, and a 37-year-old man with early degenerative arthropathy. Each case was investigated using ultrasound, revealing fusiform hypoechoic fluid collections with heterogeneous echostructure parallel to the medial gastrocnemius muscle bundle and lacking posterior reinforcement. The clinical context and ultrasound findings were critical in differentiating these cases from other conditions, such as superficial thrombophlebitis, intramuscular seroma, and intramuscular myxoma. These cases emphasize the role of ultrasound in diagnosing intramuscular dissecting Baker’s cysts. Accurate diagnosis requires careful consideration of ultrasound features in conjunction with clinical findings.

## Introduction

Baker’s cysts, typically discovered incidentally, are synovial lesions situated in the popliteal fossa. These cysts, which are neither true bursae nor true cysts [1], are distended, fluid-filled structures positioned between the medial head of the gastrocnemius and the semimembranosus tendon. This location is due to their communication with the knee joint. Research has identified two age groups with higher incidence rates: children aged four to seven years and adults aged 35 to 70 years [2].

Patients with Baker’s cysts may experience symptoms that range from acute to chronic. In acute cases, symptoms can mimic those of deep vein thrombosis, especially if the cyst ruptures. Chronic and sub-acute cases often present as a mass or pain in the popliteal fossa [1].

Baker’s cysts tend to expand in various directions, predominantly inferomedially or towards palpable superficial areas. Lateral or proximal expansion is less common [3,4]. Typically, the expansion of these cysts follows intermuscular planes [5]. However, there have been rare reports of Baker’s cysts dissecting intramuscularly. We present three cases of dissecting Baker’s cysts diagnosed by ultrasound and a literature review to assess the main differential diagnoses.

## Case presentation

Case 1

**Figure 1:**
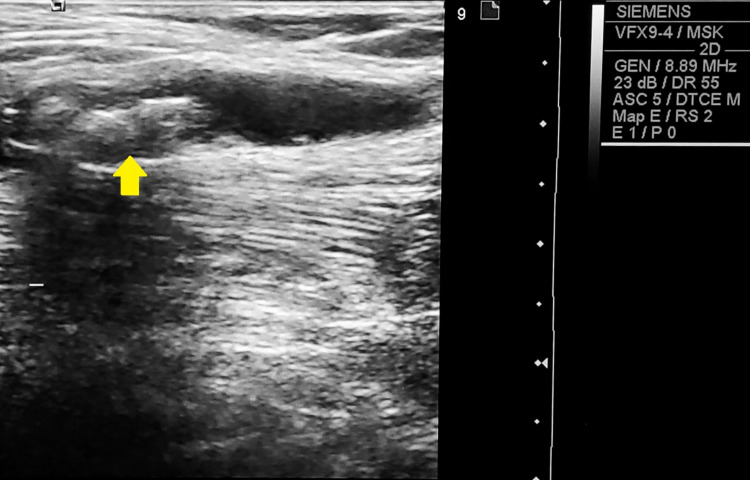
Ultrasound image from a 64-year-old woman showing a dissecting Baker’s cyst within the medial gastrocnemius muscle, featuring multiple internal calcifications (arrow).

A 64-year-old woman presented with bilateral knee pain and symptoms indicative of chronic degenerative arthropathy. Her physical examination revealed a limping gait. Knee ultrasound showed medial and lateral osteophytes at the femoral and tibial condyles and a narrowing of the medial femorotibial spaces in both knees (<3 mm), with bilateral medial meniscal extrusion (4.3 mm on the right and 5.4 mm on the left). A fusiform hypoechoic fluid collection with heterogeneous echostructure was identified in the calf, parallel to the right medial gastrocnemius muscle bundle, measuring 3.84 cm x 0.91 cm, and containing multiple internal calcifications (Figure 1). The ultrasound findings were consistent with bilateral severe gonarthrosis, bilateral medial meniscal extrusion (4.3 mm on the right and 5.4 mm on the left), and a dissecting Baker’s cyst in the right medial gastrocnemius with internal calcifications.

Case 2

**Figure 2:**
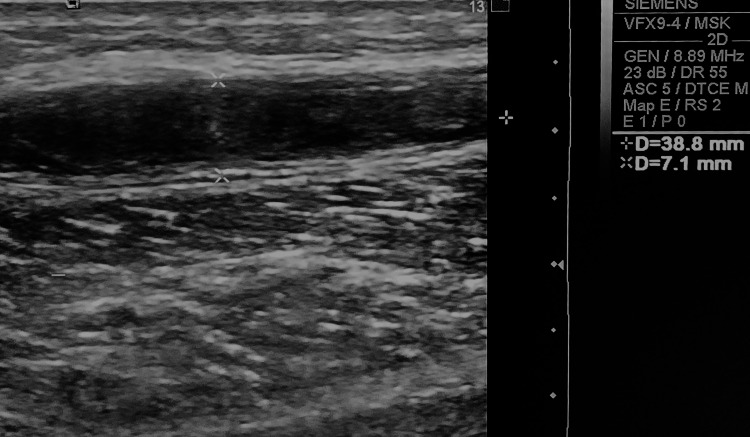
Ultrasound image from an 80-year-old man depicting a dissecting Baker’s cyst located within the medial gastrocnemius muscle.

An 80-year-old man sought evaluation for a palpable mass in the popliteal fossa and a history of a nonpainful calf mass that spontaneously regressed. Physical examination revealed a slight increase in the popliteal fossa volume. Knee ultrasound findings included a fusiform hypoechoic fluid collection with heterogeneous echostructure in the calf, parallel to the medial gastrocnemius muscle bundle, measuring 3.88 cm x 0.71 cm, without posterior reinforcement (Figure 2). Adjacent vessels appeared normal. The diagnosis was a dissecting Baker’s cyst in the medial gastrocnemius.

Case 3

**Figure 3:**
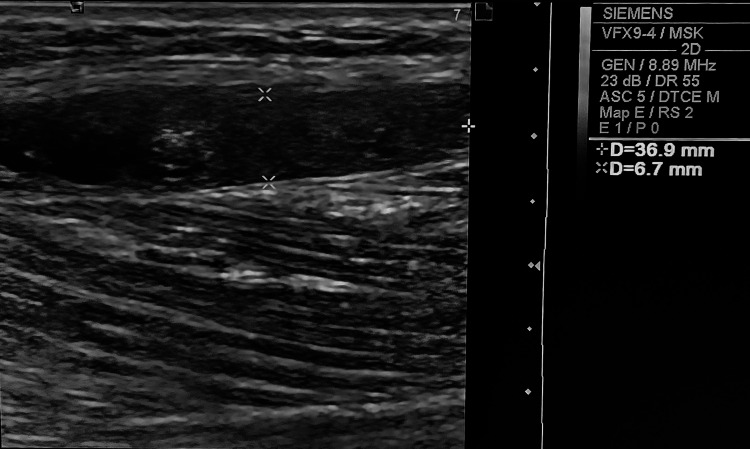
Ultrasound image from a 37-year-old individual displaying a dissecting Baker’s cyst within the medial gastrocnemius muscle.

A 37-year-old man presented with signs suggestive of early degenerative arthropathy and discomfort in the gastrocnemius. Physical examination noted an increase in volume in the knee’s suprapatellar region, with no reported trauma history. Knee ultrasound findings included a medium abundance effusion in the suprapatellar bursa and osteophytic lesions at the femoral condyles. A fusiform hypoechoic fluid collection with a heterogeneous echostructure was found in the calf, parallel to the medial gastrocnemius muscle bundle, measuring 3.69 cm x 6.7 cm, without evidence of posterior reinforcement (Figure 3). The ultrasound findings indicated gonarthrosis, associated with a medium abundance suprapatellar effusion and a dissecting Baker’s cyst in the medial gastrocnemius.

## Discussion

Baker’s cysts, which are not true cysts, arise due to direct communication between the knee joint capsule and the bursa of the medial gastrocnemius and semitendinosus. Although typically discovered incidentally, ultrasound is the first-choice diagnostic tool for these cysts. The characteristic ultrasound features of Baker’s cysts include a well-defined anechoic cystic image with a “collar” at its deepest extension located between the semimembranosus tendon and the medial head of the gastrocnemius [1]. Often considered a complication of Baker’s cysts, intramuscular dissecting Baker’s cysts usually extend inferomedially, but may also spread proximally, anteriorly, intermuscularly, or intramuscularly [1]. The pathophysiology behind the formation of intramuscularly dissecting Baker’s cysts is not fully understood. It is hypothesized that these cysts extend from the popliteus along muscular planes that offer the least resistance, with weakened muscle fascia possibly predisposing to intramuscular cystic dissection [1,5].

Rare cases of Baker’s cysts dissecting the medial gastrocnemius, as reported in the literature, were incidentally identified on magnetic resonance imaging (MRI), typically described as a cystic mass with high T2 signal extending extramuscularly up to the knee joint level [5]. The main ultrasound features of the three presented cases include a fusiform hypoechoic fluid collection with heterogeneous echostructure and regular contours aligned with the gastrocnemius muscle bundle and lacking posterior reinforcement. While intra-articular communication was not confirmed, the differential diagnoses were effectively ruled out based on clinical context and concurrent assessment of the adjacent vascular network.

**Figure 4:**
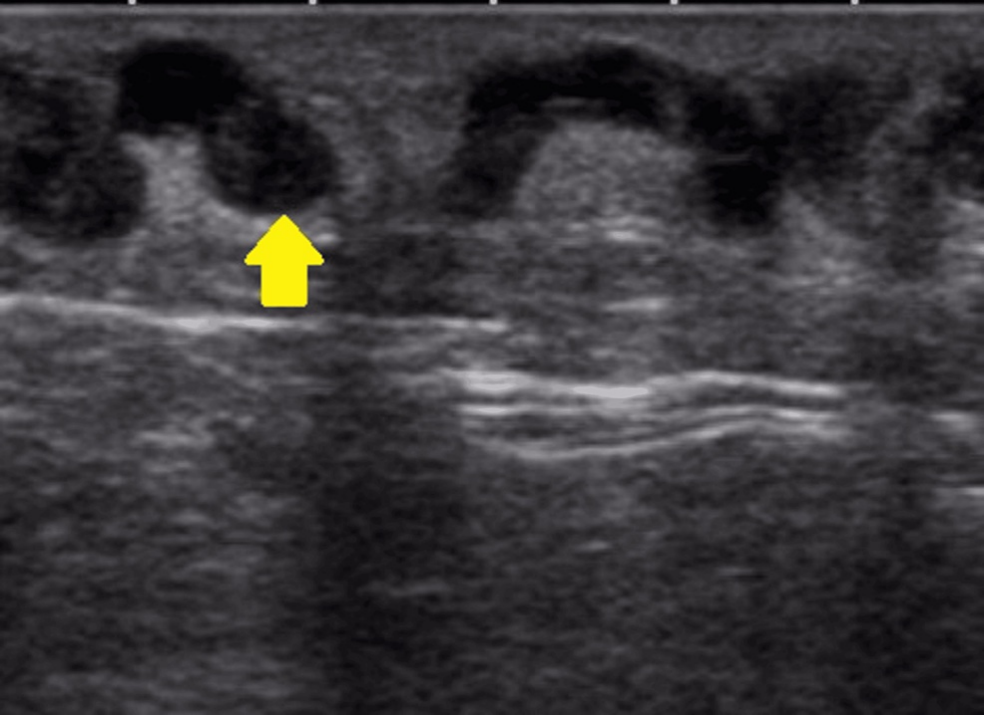
Ultrasound image illustrating superficial thrombophlebitis, indicative of thrombosis (arrow) and inflammation in the superficial veins. Case courtesy of Roberto Schubert, Radiopaedia.org, rID: 18862 [6]. This image is used under the Creative Commons Attribution-NonCommercial-ShareAlike 3.0 Unported License. This work is licensed under a Creative Commons BY-NC-SA 3.0 License.

**Figure 5:**
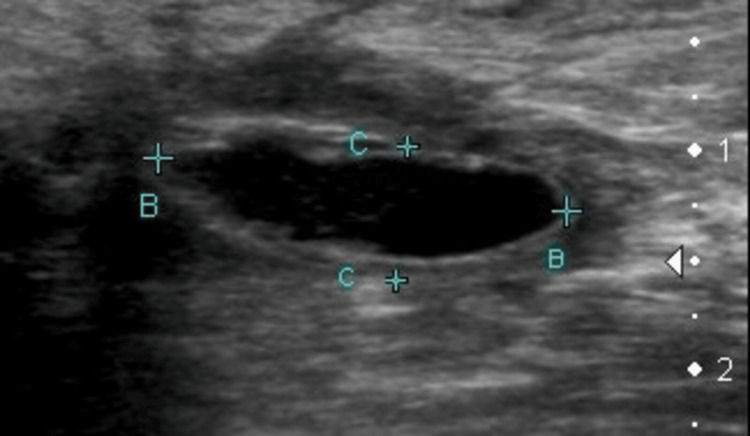
Ultrasound depiction of an intramuscular seroma showcasing its initial anechoic presentation and the potential evolution to exhibit thickened nodular margins. Case courtesy of Garth Kruger, Radiopaedia.org, rID: 18781 [8]. This image is used under the Creative Commons Attribution-NonCommercial-ShareAlike 3.0 Unported License. This work is licensed under a Creative Commons BY-NC-SA 3.0 License.

The differential diagnoses include superficial thrombophlebitis, intramuscular seroma, and intramuscular myxoma. Superficial thrombophlebitis, characterized by thrombosis in superficial veins accompanied by adjacent tissue inflammation, appears on ultrasound as a non-compressible or partially compressible hypoechoic zone within the vessel (Figure 4) [6,7]. Intramuscular seroma (Figure 5), typically a post-surgical or post-traumatic serous fluid collection, appears anechoic on ultrasound initially, potentially developing thickened nodular margins as it matures [8,9]. Intramuscular myxoma, a rare benign mesenchymal soft tissue tumor, presents as a well-circumscribed hypoechoic or anechoic mass with potential internal echoes and a heterogeneous echostructure on ultrasound. Most cases show posterior acoustic reinforcement and a characteristic sonographic bright rim sign, with a “bright cap sign” visible in some instances. These lesions are generally hypovascular or avascular on color Doppler [10-12].

Baker’s cysts are frequently associated with degenerative knee arthropathy [13]. MRI studies of Baker’s cysts reveal an association with intra-articular lesions in 87% to 98% of cases [3,14]. In our study, two cases had concurrent joint lesions: one with gonarthrosis and suprapatellar effusion, the other with severe knee osteoarthritis and medial meniscal extrusion. Notably, a 64-year-old woman with severe osteoarthritis presented with multiple internal calcifications in the cyst. Internally mobile calcifications in a Baker’s cyst are rare and may originate from the knee joint, due to causes like destructive arthropathy or may develop within the cyst itself through chondrometaplasia [15].

## Conclusions

After ruling out potential differential diagnoses in the appropriate clinical setting, any well-defined hypoechoic fusiform fluid collection parallel to the medial gastrocnemius muscle bundle, with heterogeneous echostructure and no posterior reinforcement, is likely an intramuscular dissecting Baker’s cyst. Incorporating rapid calf assessment in every knee ultrasound protocol could be beneficial, considering the high association of Baker’s cysts with joint injuries, which are common reasons for knee ultrasound examinations.
